# Efficacy and safety of small interfering RNA (siRNA) therapies for hypertriglyceridemia and mixed dyslipidemia: an updated systematic review and meta-analysis

**DOI:** 10.3389/fphar.2026.1736821

**Published:** 2026-01-26

**Authors:** Yifan Gao, Yanmin Bai, Xu Mu, Xingxue Pang

**Affiliations:** 1 Dongzhimen Hospital, Beijing University of Chinese Medicine, Beijing, China; 2 Third Department of Cardiology, Dongzhimen Hospital, Beijing University of Chinese Medicine, Beijing, China

**Keywords:** hypertriglyceridemia, meta-analysis, mixed dislipidemia, small interfering RNA, solbinsiran

## Abstract

**Background:**

Hypertriglyceridemia (HTG) and mixed dyslipidemia are significant risk factors for cardiovascular diseases. Despite the widespread use of traditional therapies, many patients continue to experience elevated triglycerides and residual cardiovascular risk. Small interfering RNA (siRNA) therapies represent a novel approach to lipid-lowering treatment.

**Methods:**

A systematic review and meta-analysis were conducted on randomized controlled trials comparing siRNA versus placebo for hypertriglyceridemia or mixed dyslipidemia. The search included PubMed, Cochrane Library, Web of Science, and Embase databases from inception to 1 October 2025, limited to English-language publications. Data extraction was performed independently by two authors.

**Results:**

Eight RCTs involving 2,671 participants met the inclusion criteria. siRNA therapies significantly reduced triglycerides (TG) (MD, −52%; 95%, −57.9 to −46.2), non-high-density lipoprotein cholesterol (non-HDL-C) (MD, −21.9%; 95%, −26 to −17.7), very low-density lipoprotein cholesterol (VLDL-C) (MD, −49.5%; 95%, −60.1 to −38.9), apolipoprotein B (apoB) (MD, −12.6%; 95%, −16.4 to −8.8), and remnant cholesterol (MD, −64.8%; 95%, −81.7 to −47.9)compared with placebo. The reduction in TG was particularly notable. Subgroup analysis revealed that ANGPTL3-targeted therapies resulted in more substantial reductions in low-density lipoprotein cholesterol (MD, −13.2%; 95% CI, −20.1 to −6.2), while APOC3-targeted therapies had a neutral effect on LDL-C levels (MD, 0.6%; 95% CI, −5.7–6.9) (*p* for interaction = 0.00001). On the other hand, APOC3-targeted therapies significantly increased high-density lipoprotein cholesterol levels (MD, 40.9%; 95% CI, 31.6–50.2), whereas ANGPTL3-targeted therapies led to a reduction in HDL-C levels (MD, −20.2%; 95% CI, −25.4 to −14.9) (p for interaction = 0.00001). No significant differences were observed in the risk of adverse events between siRNA therapy and placebo (RR, 1.02; 95% CI, 0.96–1.09).

**Conclusion:**

siRNA therapies demonstrate significant efficacy in reducing triglycerides and improving lipid profiles in patients with HTG and mixed dyslipidemia. APOC3-targeted treatments primarily reduce triglycerides while increasing HDL-C, whereas ANGPTL3-targeted therapies offer broader lipid modulation, including substantial reductions in LDL-C. Both therapies demonstrate favorable safety profiles.

## 1 Introduction

Hypertriglyceridemia (HTG) and mixed dyslipidemia are highly prevalent among individuals with metabolic syndrome, type 2 diabetes, obesity, and nonalcoholic fatty liver disease, and are recognized risk factors for atherosclerotic cardiovascular disease (ASCVD), nonalcoholic steatohepatitis, and acute pancreatitis (Gaudet et al., 2024; Zhou et al., 2022; Kessler et al., 2025; Nawaz et al., 2015). Epidemiologic studies indicate that the adult prevalence of HTG at varying severities can reach double-digit levels (Laufs et al., 2020). Importantly, even under statin therapy achieving low-density lipoprotein cholesterol (LDL-C) control, substantial residual cardiovascular risk persists, largely attributable to elevated triglycerides (TG) and triglyceride-rich lipoprotein (TRL) remnants, including non–HDL-C and apolipoprotein B (apoB) (Watts et al., 2023; Vasas et al., 2024; Hussain et al., 2020; Malick et al., 2023a).

Current management strategies for HTG and mixed dyslipidemia provide limited and inconsistent benefits in TG lowering and cardiovascular outcomes, underscoring the need for more effective and durable approaches targeting TRL and apoB-containing lipoproteins (Filtz et al., 2024; Berglund et al., 2012; Chapman et al., 2011). Small interfering RNA (siRNA) therapeutics offer a novel strategy by selectively silencing hepatocyte-expressed genes involved in TG metabolism through RNA-induced silencing complex–mediated mRNA degradation. GalNAc conjugation enables selective uptake of siRNA by hepatocytes through the asialoglycoprotein receptor, resulting in more efficient liver targeting and improved potency and safety (Katzmann et al., 2020; Gao et al., 2023; Masson et al., 2024; Ward et al., 2022). Among patients with HTG and mixed dyslipidemia, siRNA therapies targeting APOC3 and ANGPTL3 have demonstrated marked TG reductions and favorable effects on non–HDL-C, apoB, and very-low-density lipoprotein cholesterol (VLDL-C) across phase I–III clinical trials (Gaudet et al., 2024; Huynh, 2024; Ray et al., 2025; Bornfeldt, 2024; Schwabe et al., 2020; Lim, 2024). Compared with earlier antisense oligonucleotide approaches, siRNAs are associated with less frequent dosing and improved platelet safety (Tang et al., 2024).

Despite these promising results, substantial heterogeneity exists across trials with respect to patient populations, background lipid-lowering therapies, follow-up duration, outcome definitions, and safety assessments. Moreover, a previous meta-analysis (Kamrul-Hasan et al., 2024) did not incorporate recently published trials, including emerging data on ANGPTL3-targeting agents such as solbinsiran, and insufficiently explored whether lipid-lowering effects differ between siRNA therapies targeting APOC3 and ANGPTL3. Clarifying these potential target-specific differences is important for personalized treatment strategies. Accordingly, this study aimed to provide an updated synthesis of the evidence on siRNA therapies for hypertriglyceridemia and mixed dyslipidemia, with particular attention to potential target-specific differences.

## 2 Methods

### 2.1 Protocol and registration

The protocol for this systematic review and meta-analysis was prospectively registered in the International Prospective Systematic Review Registry (PROSPERO; CRD420251151154). This study was conducted and reported according to the Preferred Reporting Items for Systematic Reviews and Meta-Analyses (PRISMA) reporting guidelines (Page et al., 2021).

### 2.2 Data sources and searches

The search was conducted across four databases: PubMed, EMBASE, Cochrane Library, and Web of Science, covering publications from their inception to 1 October 2025. Terms included “hypertriglyceridemia,” “mixed dyslipidemia,” “small interfering RNA,” “Solbinsiran,” “Olezarsen,” “Plozasiran,” “Zodasiran,” and others. Additional searches were performed before the final analysis, and relevant studies were included. For detailed search strategies, please refer to the Supplementary Table S1.

### 2.3 Eligibility criteria and study selection

Studies meeting the following criteria were considered eligible for inclusion: (1) Study type: RCT studies; (2) Patients with a baseline diagnosis of hypertriglyceridemia or mixed dyslipidemia; (3) Exposure: treatment with small interfering RNA (siRNA)–based therapies (antisense oligonucleotide therapies were excluded); Control: placebo; (4) Outcomes: percentage change from baseline in lipid parameters, including triglycerides, LDL-C, HDL-C, non-HDL-C, VLDL-C, apoB and remnant cholesterol. The study selection process was as follows: (1) Exclude duplicate publications; (2) Read the study titles and abstracts, excluding reviews, conference abstracts, letters, pharmacokinetic studies, animal studies, protocols, and completely unrelated studies; (3) Read the full text, excluding studies that did not meet the intervention and population criteria, Phase I trials, non-RCTs, or studies without data. Two independent reviewers (YB and XM) screened titles and abstracts based on inclusion criteria. Any discrepancies in the search and selection process were resolved through consultation with a third reviewer (YG). If a study appeared to meet the inclusion criteria, the full text was retrieved for further assessment.

### 2.4 Data extraction

After identifying the studies to be included, two reviewers independently extracted the data. The data extracted from each study included: (1) General information, including study title, publication year, number of centers, study design, population, sample size, duration of study, interventions, reported outcomes, and the definition of hypertriglyceridemia and mixed dyslipidemia; (2) Baseline information, including gender, age, lipid levels, percentage of statin use, percentage of diabetes, and percentage of chronic kidney disease. The reviewers retrieved relevant information from the manuscript text, tables, figures, and Supplementary Material. If detailed information was not provided in the article, we used GetData Graph Digitizer to measure graphical data. Discrepancies were resolved through discussion with a third reviewer (YG).

### 2.5 Outcomes and definitions

The primary outcome of this study is the percentage change in triglycerides from baseline. The secondary outcomes include the percentage changes from baseline in other lipid parameters (including LDL-C, HDL-C, non-HDL-C, VLDL-C, apoB, and remnant cholesterol). Safety outcomes include adverse events. Hypertriglyceridemia is defined as an abnormal elevation of triglyceride levels in the blood, typically referring to a fasting plasma triglyceride concentration greater than 150 mg/dL (1.7 mmol/L). Mixed dyslipidemia is defined as triglyceride levels between 150 and 499 mg/dL, with LDL-C levels ≥70 mg/dL or non-high-density lipoprotein cholesterol levels ≥100 mg/dL.

### 2.6 Risk of bias and certainty of evidence

The reviewers (YB and XP) independently assessed the risk of bias using version two of the Cochrane Collaboration Risk of Bias tool (Sterne et al., 2019). The trials were evaluated for bias risk in the following domains: randomization process, deviations from intended interventions, missing outcome data, measurement of outcomes, and selection of reported results. It is recommended to use a graded assessment, development, and evaluation framework to assess the certainty of the evidence.

### 2.7 Statistical analysis

#### 2.7.1 Data synthesis

Our meta-analysis rigorously assessed the clinical and methodological heterogeneity of the included studies, focusing on differences in patient populations, intervention protocols, and outcome measurements. Statistical heterogeneity was evaluated using the χ^2^ homogeneity test and the I^2^ statistic. Considering the observed between-study heterogeneity, a random-effects model was applied to account for variability across studies.

For continuous outcomes, the random-effects inverse variance method was used to estimate the pooled effects of small interfering RNA (siRNA) therapy on percentage changes in TG, LDL-C, VLDL-C, HDL-C, non-HDL-C, apoB, and remnant cholesterol relative to baseline, with results summarized as mean differences (MDs) with 95% CIs and standard deviations (SDs). For dichotomous outcomes, including adverse events, serious adverse events, and injection site reactions, the random-effects Mantel–Haenszel method was used, and results were reported as risk ratios (RRs) with 95% confidence intervals (CIs).

Leave-one-out sensitivity analyses were conducted to evaluate the influence of individual studies on the overall effect estimates. We carried out meta-regressions analyzing if moderating variables such as target and specific siRNA drug caused heterogeneity for outcomes with substantial heterogeneity (I^2^ ≥ 50%) and at least five included studies. Meta-regression was not performed for outcomes with fewer studies due to insufficient statistical power.

Due to the small number of included studies, publication bias was not formally assessed using funnel plots or asymmetry tests, as these methods have low statistical power under such conditions (Sterne et al., 2011).

All statistical analyses were conducted using Stata 18 software, with *p*-values <0.05 considered statistically significant.

#### 2.7.2 Subgroup analysis based on target ANGPTL3 vs. APOC3

The results of the pre-specified subgroup analysis were stratified for the ANGPTL3 and APOC3 subgroups. The interaction p-value was calculated to test the statistical significance of differences between subgroups. An interaction *p*-value <0.01 was considered significant (Sun et al., 2010).

## 3 Results

**FIGURE 1:**
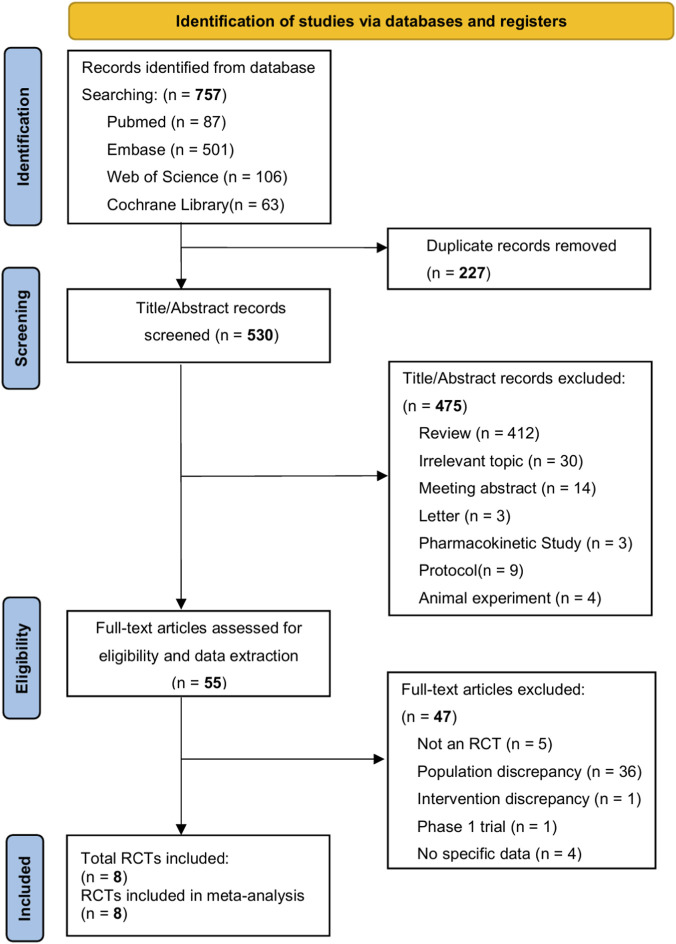
Flowchart of study selection.

The PRISMA flow diagram for the study selection process is shown in Figure 1. A total of 757 records were identified through electronic database searches. After removing 227 duplicate records and screening titles and abstracts, 55 full-text articles were retrieved. Ultimately, eight studies met the inclusion criteria and were included in the meta-analysis (Gaudet et al., 2024; Ray et al., 2025; Tardif et al., 2022; Bergmark et al., 2024; Stroes et al., 2024; Ballantyne et al., 2024; Rosenson et al., 2024; Bergmark et al., 2025).

### 3.1 Included studies

A total of eight studies evaluated the percentage changes in lipid levels from baseline in patients with hypertriglyceridemia or mixed dyslipidemia receiving small interfering RNA (siRNA) therapy compared to those receiving a placebo (Gaudet et al., 2024; Ray et al., 2025; Tardif et al., 2022; Bergmark et al., 2024; Stroes et al., 2024; Ballantyne et al., 2024; Rosenson et al., 2024; Bergmark et al., 2025). In these studies, all eight used a matched placebo in the control group. In the intervention group, two studies targeted ANGPTL3 (Tardif et al., 2022; Rosenson et al., 2024), while six studies targeted APOC3 (Gaudet et al., 2024; Ray et al., 2025; Bergmark et al., 2024; Stroes et al., 2024; Ballantyne et al., 2024; Bergmark et al., 2025). Most or all participants in the included RCTs were taking lipid-lowering medications, with a balanced proportion of participants using lipid-lowering drugs across the groups. Detailed intervention information and background medication are provided in Supplementary Table S2.

### 3.2 Baseline characteristics

A summary of the study characteristics is provided in Table 1 and Supplementary Table S2. Our systematic review and meta-analysis included 2,671 individuals from eight different studies. Of these, 2,000 individuals (75%) were assigned to the siRNA intervention group, while the remaining 671 individuals (25%) were assigned to the placebo group. The average age of the participants was 60.1 years (SD: 10.8 years), with 59.4% (1,587) male and 40.6% (1,084) female. Over 80% of the participants were primarily treated with statins, at least 21% with fibrates, and at least 16.2% with Omega-3 fatty acids.

**TABLE 1:** Characteristics of included trials.

Trial ID	Trial design	Duration of study	No. of patients	Study population	Primary endpoint	siRNA drug (target)	Age mean (SD), y	Male sex, n (%)	Baseline LDL-C, mean (SD), mg/dL	Baseline TG, mean (SD), mg/dL
ARCHES-2 2024	Prospective, randomized, double-blind, placebo-controlled, dose-ranging phase 2b trial	36 weeks	204	Mixed hyperlipidemia	Percent change from baseline to week 24 in fasting triglyceride levels	Zodasiran (ANGPTL3)	60.5 (11.6)	109 (53.4)	97.5 (35.6)	246.1 (89.4)
MUIR 2024	Prospective, randomized, double-blind, placebo-controlled, phase 2b trial	48 weeks	353	Mixed hyperlipidemia	Percent change in fasting triglyceride level at week 24	Plozasiran (APOC3)	60.7 (11)	199 (56.4)	103.3 (38.2)	244.1 (78.3)
PROLONG-ANG3 2025	Prospective, randomized, double-blind, placebo-controlled, phase 2 trial	360 days	205	Mixed hyperlipidemia	Percent change in fasting triglyceride from baseline to day 180	Solbinsiran (ANGPTL3)	56.7 (11.3)	94 (45.9)	123.3 (37.5)	237.5 (91.1)
Balance 2024	Prospective, randomized, double-blind, placebo-controlled, phase 3 trial	53 weeks	66	Hypertriglyceridemia	Percent change in triglyceride from baseline to 6 months	Olezarsen (APOC3)	45 (13.4)	28 (42.4)	19 (10.9)	2,630 (1,316)
Bridge–TIMI 73a 2024	Prospective, randomized, double-blind, placebo-controlled, dose-ranging phase 2b trial	15 months	154	Hypertriglyceridemia	Percent change in triglyceride from baseline to 6 months	Olezarsen (APOC3)	62.4 (11.7)	89 (57.8)	82.4 (32.7)	253 (110)
ESSENCE–TIMI 73b 2025	Prospective, randomized, double-blind, placebo-controlled, phase 3 trial	15 months	1,349	Hypertriglyceridemia	Percent change in triglyceride from baseline to 6 months	Olezarsen (APOC3)	63.2 (9.1)	806 (59.7)	84.6 (37.1)	245 (87)
NCT03385239 2022	Prospective, randomized, double-blind, placebo-controlled, dose-ranging phase 2 trial	15 months	114	Hypertriglyceridemia	Percent change in fasting triglyceride from baseline to 6 months	Olezarsen (APOC3)	65.3 (8.1)	86 (75.4)	68.9 (25.2)	284 (85)
SHASTA-2 2024	Prospective, randomized, double-blind, placebo-controlled, dose-ranging phase 2b trial	48 weeks	226	Hypertriglyceridemia	Percent change in triglyceride level at week 24	Plozasiran (APOC3)	55 (11)	176 (78)	72 (41)	897 (625)

### 3.3 Percent change in TG

**FIGURE 2:**
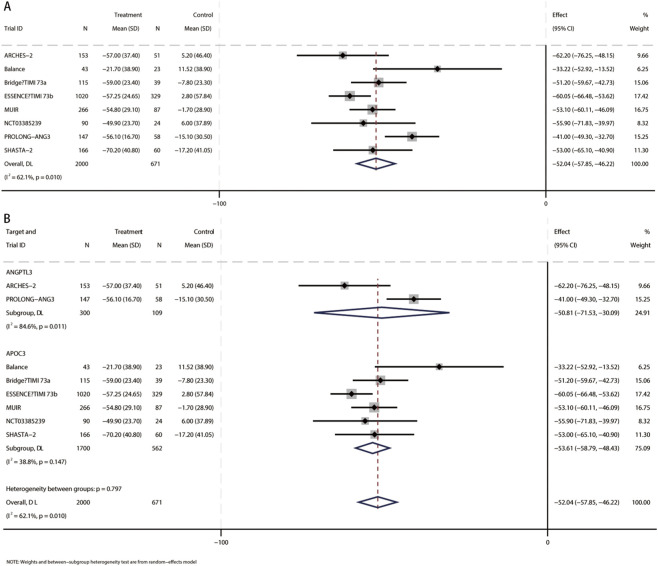
Forest plots for percent changes in triglycerides (TG) levels. **(A)** Overall analysis of siRNA therapy versus placebo. **(B)** Subgroup analysis by siRNA target (ANGPTL3 vs. APOC3).

In a meta-analysis comprising eight RCTs (Gaudet et al., 2024; Ray et al., 2025; Tardif et al., 2022; Bergmark et al., 2024; Stroes et al., 2024; Ballantyne et al., 2024; Rosenson et al., 2024; Bergmark et al., 2025), 2,671 participants (mean [SD] age, 61.1 [10.8] years; 1,587 male [59.4%]) were included. Compared with placebo, the pooled mean difference in triglycerides was −52% (95% CI, −57.9 to −46.2; I^2^ = 62.1%), indicating a statistically significant reduction in TG with small interfering RNA therapy (Figure 2A). These studies included patients using siRNA targeting ANGPTL3 (409 participants, mean [SD] age, 58.6 [11.6] years, 203 male [49.6%]) and patients using siRNA targeting APOC3 (2,262 participants, mean [SD] age 61.5 [10.6] years, 1,384 male [61.2%]). Compared with placebo, triglyceride levels were significantly reduced in patients receiving siRNA targeting ANGPTL3 (MD, −50.8%; 95% CI, −71.5 to −30.1), and triglyceride levels were also significantly reduced in patients receiving siRNA targeting APOC3 (MD, −53.6%; 95% CI, −58.8 to −48.4) (Figure 2B). No interaction was found (*p* for interaction = 0.8).

### 3.4 Percent change in LDL-C

**FIGURE 3:**
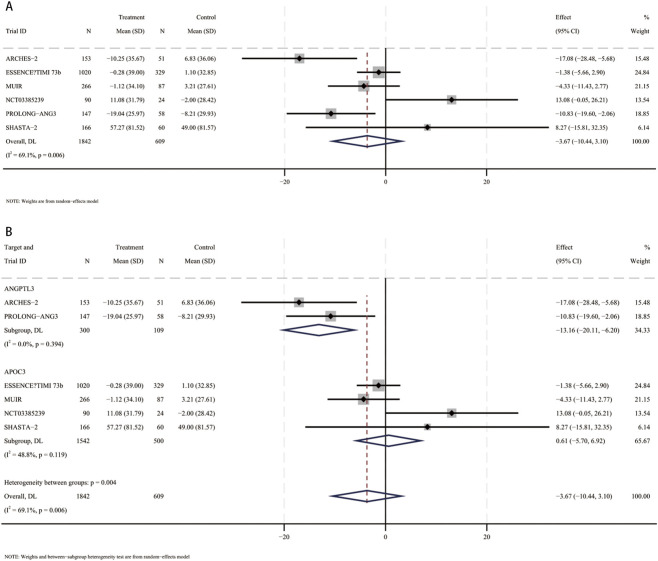
Forest plots for percent changes in low-density lipoprotein cholesterol (LDL-C) levels. **(A)** Overall analysis of siRNA therapy versus placebo. **(B)** Subgroup analysis by siRNA target (ANGPTL3 vs. APOC3).

In a meta-analysis comprising six RCTs (Gaudet et al., 2024; Ray et al., 2025; Tardif et al., 2022; Ballantyne et al., 2024; Rosenson et al., 2024; Bergmark et al., 2025), 2,451 participants (mean [SD] age, 61.4 [10.4] years; 1,470 male [60%]) were included. Compared with placebo, the pooled mean difference in LDL-C was −3.7% (95% CI, −10.4 to 3.1; I^2^ = 69.1%), indicating a not statistically significant reduction in LDL-C with small interfering RNA therapy (Figure 3A). These studies included patients using siRNA targeting ANGPTL3 (409 participants, mean [SD] age, 58.6 [11.6] years, 203 male [49.6%]) and patients using siRNA targeting APOC3 (2042 participants, mean [SD] age 70 [10] years, 1,267 male [62%]). Compared with placebo, LDL-C levels were significantly reduced in patients receiving siRNA targeting ANGPTL3 (MD, −13.2%; 95% CI, −20.1 to −6.2), but LDL-C levels were not significantly reduced in patients receiving siRNA targeting APOC3 (MD, 0.6%; 95% CI, −5.7–6.9) (Figure 3B). Interaction analysis showed that there was an interaction between the APOC3 and ANGPTL3 targets in the association between small interfering RNA therapy and the percentage change in LDL-C (*p* for interaction = 0.003).

### 3.5 Percent change in HDL-C

**FIGURE 4:**
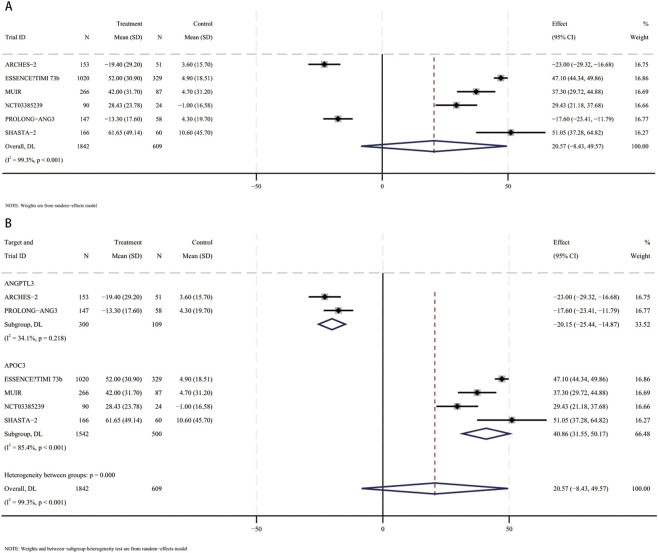
Forest plots for percent changes in high-density lipoprotein cholesterol (HDL-C) levels. **(A)** Overall analysis of siRNA therapy versus placebo. **(B)** Subgroup analysis by siRNA target (ANGPTL3 vs. APOC3).

In a meta-analysis comprising six RCTs (Gaudet et al., 2024; Ray et al., 2025; Tardif et al., 2022; Ballantyne et al., 2024; Rosenson et al., 2024; Bergmark et al., 2025), 2,451 participants (mean [SD] age, 61.4 [10.4] years; 1,470 male [60%]) were included. Compared with placebo, the pooled mean difference in HDL-C was 20.6% (95% CI, −8.4 to 49.6; I^2^ = 99.3%), indicating a not statistically significant increase in HDL-C with small interfering RNA therapy (Figure 4A). These studies included patients using siRNA targeting ANGPTL3 (409 participants, mean [SD] age, 58.6 [11.6] years, 203 male [49.6%]) and patients using siRNA targeting APOC3 (2042 participants, mean [SD] age 70 [10] years, 1,267 male [62%]). Compared with placebo, HDL-C levels were significantly reduced in patients receiving siRNA targeting ANGPTL3 (MD, −20.2%; 95% CI, −25.4 to −14.9). In contrast, patients receiving siRNA therapy targeting APOC3 had a significant increase in HDL-C levels (MD, 40.9%; 95% CI, 31.6–50.2) (Figure 4B). Interaction analysis showed that there was an interaction between the APOC3 and ANGPTL3 targets in the association between small interfering RNA therapy and the percentage change in HDL-C (*p* for interaction = 0.00001).

### 3.6 Percent change in non-HDL-C

**FIGURE 5:**
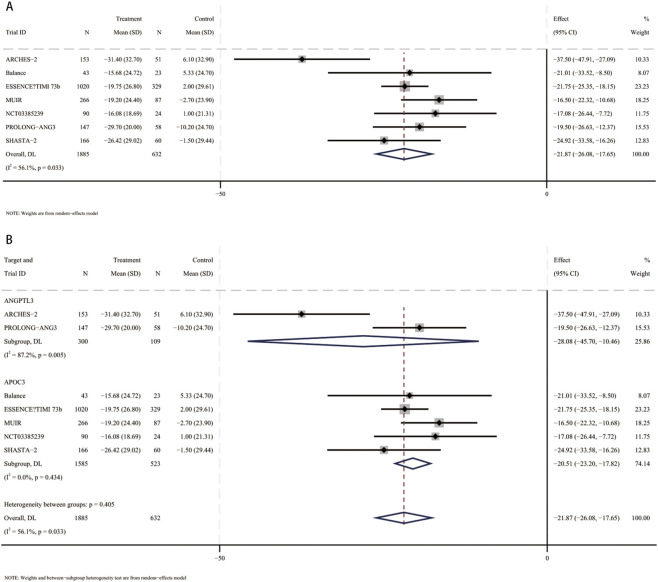
Forest plots for percent changes in non-high-density lipoprotein cholesterol (Non-HDL-C) levels. **(A)** Overall analysis of siRNA therapy versus placebo. **(B)** Subgroup analysis by siRNA target (ANGPTL3 vs. APOC3).

In a meta-analysis comprising seven RCTs (Gaudet et al., 2024; Ray et al., 2025; Tardif et al., 2022; Stroes et al., 2024; Ballantyne et al., 2024; Rosenson et al., 2024; Bergmark et al., 2025), 2,517 participants (mean [SD] age, 61 [10.8] years; 1,498 male [59.5%]) were included. Compared with placebo, the pooled mean difference in Non-HDL-C was −21.9% (95% CI, −26.1 to −17.6; I^2^ = 56.1%), indicating a statistically significant reduction in Non-HDL-C with small interfering RNA therapy (Figure 5A). These studies included patients using siRNA targeting ANGPTL3 (409 participants, mean [SD] age, 58.6 [11.6] years, 203 male [49.6%]) and patients using siRNA targeting APOC3 (2,108 participants, mean [SD] age 61.4 [10.5] years, 1,295 male [61.4%]). Compared with placebo, HDL-C levels were significantly reduced in patients receiving siRNA targeting ANGPTL3 (MD, −28.1%; 95% CI, −45.7 to −10.5), and non-HDL-C levels were also significantly reduced in patients receiving siRNA targeting APOC3 (MD, −20.5%; 95% CI, −23.2 to −17.8) (Figure 5B). No interaction was found (*p* for interaction = 0.4).

### 3.7 Percent change in VLDL-C

**FIGURE 6:**
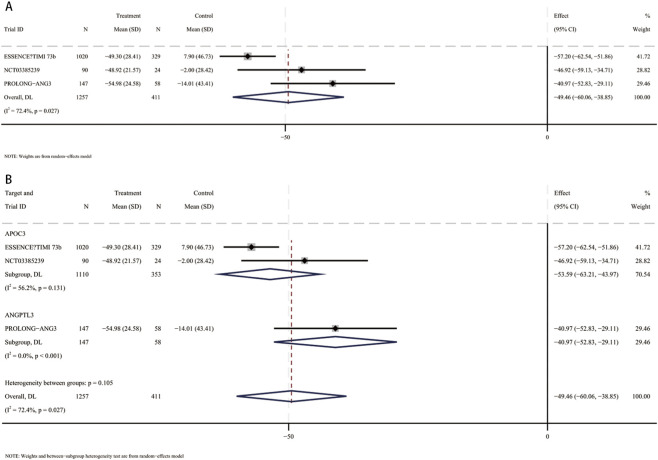
Forest plots for percent changes in very low-density lipoprotein cholesterol (VLDL-C) levels. **(A)** Overall analysis of siRNA therapy versus placebo. **(B)** Subgroup analysis by siRNA target (ANGPTL3 vs. APOC3).

In a meta-analysis comprising three RCTs (Ray et al., 2025; Tardif et al., 2022; Bergmark et al., 2025), 1,668 participants (mean [SD] age, 62.5 [9.6] years; 986 male [59.1%]) were included. Compared with placebo, the pooled mean difference in VLDL-C was −49.5% (95% CI, −60.1 to −38.9; I^2^ = 72.4%), indicating a statistically significant reduction in VLDL-C with small interfering RNA therapy (Figure 6A). These studies included patients using siRNA targeting ANGPTL3 (205 participants, mean [SD] age, 56.7 [11.3] years, 94 male [45.9%]) and patients using siRNA targeting APOC3 (1,463 participants, mean [SD] age 63.4 [9] years, 892 male [61%]). Compared with placebo, VLDL-C levels were significantly reduced in patients receiving siRNA targeting ANGPTL3 (MD, −41%; 95% CI, −52.8 to −29.1), and VLDL-C levels were also significantly reduced in patients receiving siRNA targeting APOC3 (MD, −53.6%; 95% CI, −63.2 to −44) (Figure 6B). No interaction was found (p for interaction = 0.105).

### 3.8 Percent change in apoB

**FIGURE 7:**
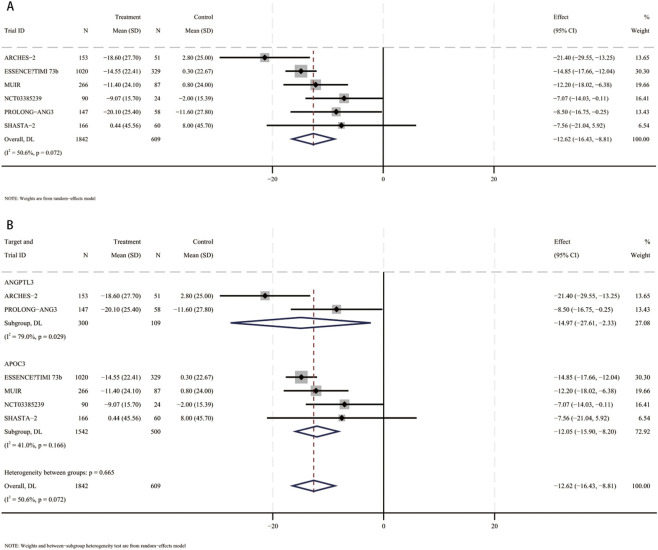
Forest plots for percent changes in apolipoprotein B (apoB) levels. **(A)** Overall analysis of siRNA therapy versus placebo. **(B)** Subgroup analysis by siRNA target (ANGPTL3 vs. APOC3).

In a meta-analysis comprising six RCTs (Gaudet et al., 2024; Ray et al., 2025; Tardif et al., 2022; Ballantyne et al., 2024; Rosenson et al., 2024; Bergmark et al., 2025), 2,451 participants (mean [SD] age, 61.4 [10.4] years; 1,470 male [60%]) were included. Compared with placebo, the pooled mean difference in apoB was −12.6% (95% CI, −16.4 to −8.8; I^2^ = 50.6%), indicating a statistically significant reduction in apoB with small interfering RNA therapy (Figure 7A). These studies included patients using siRNA targeting ANGPTL3 (409 participants, mean [SD] age, 58.6 [11.6] years, 203 male [49.6%]) and patients using siRNA targeting APOC3 (2042 participants, mean [SD] age 70 [10] years, 1,267 male [62%]). Compared with placebo, apoB levels were significantly reduced in patients receiving siRNA targeting ANGPTL3 (MD, −15%; 95% CI, −27.6 to −2.3), and apoB levels were also significantly reduced in patients receiving siRNA targeting APOC3 (MD, −12.1%; 95% CI, −15.9 to −8.2) (Figure 6B). No interaction was found (*p* for interaction = 0.7).

### 3.9 Percent change in remnant cholesterol

**FIGURE 8:**
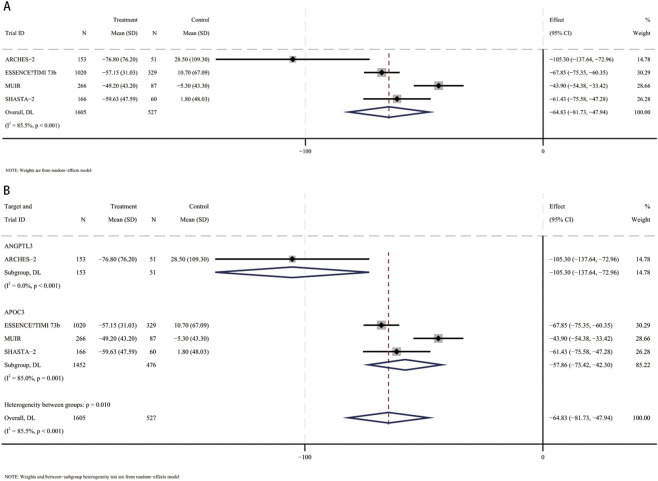
Forest plots for percent changes in remnant cholesterol levels. **(A)** Overall analysis of siRNA therapy versus placebo. **(B)** Subgroup analysis by siRNA target (ANGPTL3 vs. APOC3).

In a meta-analysis comprising four RCTs (Gaudet et al., 2024; Ballantyne et al., 2024; Rosenson et al., 2024; Bergmark et al., 2025), 2,132 participants (mean [SD] age, 61.7 [10.2] years; 1,470 male [60.5%]) were included. Compared with placebo, the pooled mean difference in remnant cholesterol was −64.8% (95% CI, −81.7 to −47.9; I^2^ = 85.5%), indicating a statistically significant reduction in remnant cholesterol with small interfering RNA therapy (Figure 8A). These studies included patients using siRNA targeting ANGPTL3 (204 participants, mean [SD] age, 60.5 [11.6] years, 109 male [53.4%]) and patients using siRNA targeting APOC3 (1,928 participants, mean [SD] age 61.8 [10.1] years, 1,181 male [61.2%]). Compared with placebo, remnant cholesterol levels were significantly reduced in patients receiving siRNA targeting ANGPTL3 (MD, −105.3%; 95% CI, −137.6 to −73) and remnant cholesterol levels were also significantly reduced in patients receiving siRNA targeting APOC3 (MD, −57.9%; 95% CI, −73.4 to −42.3) (Figure 8B). Interaction analysis showed that there was an interaction between the APOC3 and ANGPTL3 targets in the association between small interfering RNA therapy and the percentage change in remnant cholesterol (*p* for interaction = 0.01).

### 3.10 Adverse events and tolerability

**FIGURE 9:**
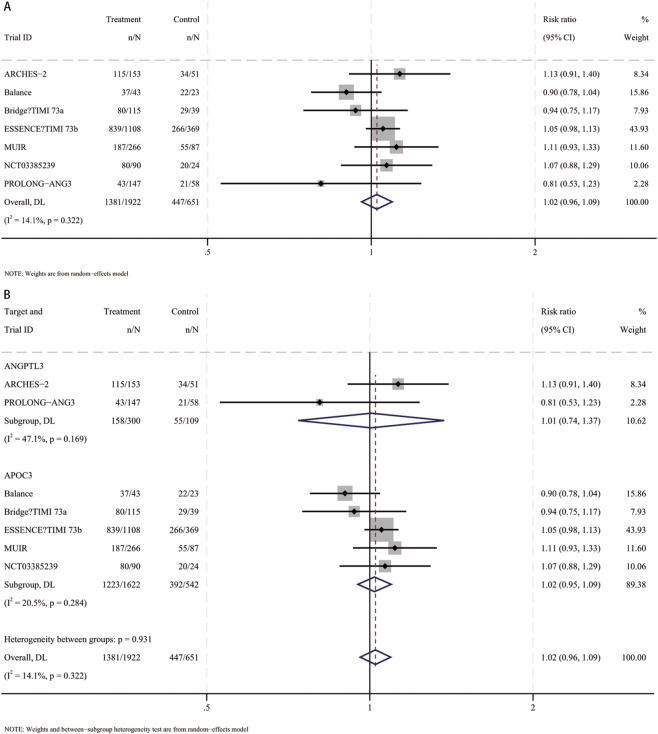
Forest plots for adverse events. **(A)** Overall analysis of siRNA therapy versus placebo. **(B)** Subgroup analysis by siRNA target (ANGPTL3 vs. APOC3).

In a meta-analysis of seven RCTs (Ray et al., 2025; Tardif et al., 2022; Bergmark et al., 2024; Stroes et al., 2024; Ballantyne et al., 2024; Rosenson et al., 2024; Bergmark et al., 2025), siRNA therapy was not associated with an increased risk of adverse events versus placebo (RR, 1.02; 95% CI, 0.96–1.09) (Figure 9A). This was consistent for agents targeting ANGPTL3 (RR, 1.01; 95% CI, 0.74–1.37) and APOC3 (RR, 1.02; 95% CI, 0.95–1.09), indicating an overall favorable tolerability profile (Figure 9B).

### 3.11 Sensitivity analysis and meta-regression

Leave-one-out sensitivity analysis showed that excluding any single study did not materially change the overall estimates of siRNA efficacy and safety relative to placebo (Supplementary Figure S1). Meta-regression analysis showed that both the target (*p* = 0.001) and the specific siRNA drug (*p* = 0.033) were associated with the intervention effect on HDL-C, while no significant associations were observed for other outcomes (all *p* > 0.05; Supplementary Table S3).

### 3.12 Study quality

Study quality is summarized in Supplementary Figure S2. Overall, our systematic review and meta-analysis revealed minimal concerns regarding the quality of the included studies. A comprehensive assessment of all studies demonstrated a low risk of bias across critical domains, including randomization, allocation concealment, intervention adherence, completeness of outcome data, accuracy and impartiality of outcome measurements, and transparency in outcome reporting. This indicates that the included clinical trials were conducted with high methodological quality, thereby enhancing the reliability of the findings (Supplementary Figure S2).

## 4 Discussion

### 4.1 Main findings

This systematic review and meta-analysis demonstrates that siRNA therapies targeting triglyceride metabolism substantially reduce triglycerides, triglyceride-rich lipoproteins, apolipoprotein B, non–HDL-C, VLDL-C, and remnant cholesterol in patients with hypertriglyceridemia and mixed dyslipidemia. Target-specific differences were observed: ANGPTL3-directed agents reduced LDL-C and modestly decreased HDL-C, whereas APOC3-directed agents showed neutral LDL-C and a tendency to increase HDL-C. Overall, these therapies were well tolerated, with no apparent increase in adverse events.

### 4.2 Interpretation of the mechanism

siRNA therapies lower triglyceride-rich lipoproteins through liver-targeted silencing of key regulatory genes (Pérez-Carrión et al., 2024; Rondinone, 2008; de Paula Brandão et al., 2020; Tian et al., 2025; Tsimikas, 2018).

Hypertriglyceridemia is a biologically heterogeneous condition arising from complex interactions between genetic predisposition and metabolic regulation. As summarized by Scicchitano et al., both rare monogenic defects affecting key components of the lipoprotein lipase pathway and more common polygenic susceptibility contribute to impaired triglyceride-rich lipoprotein clearance and variable clinical phenotypes. Regulatory proteins such as APOC3 and ANGPTL3 play central roles in modulating lipoprotein lipase activity, hepatic lipoprotein production, and remnant metabolism, providing a strong biological rationale for target-specific therapeutic strategies (Scicchitano et al., 2024).

APOC3 inhibition primarily enhances peripheral clearance of triglyceride-rich lipoproteins without substantially affecting hepatic VLDL production or LDL metabolism, explaining neutral LDL-C effects and a tendency toward higher HDL-C levels (Malick et al., 2023b; Zhang, 2021; Visser et al., 2022; Khetarpal et al., 2017; Chen et al., 2024). In contrast, ANGPTL3 inhibition accelerates triglyceride-rich lipoprotein processing and reduces LDL precursors while enhancing HDL catabolism, accounting for concurrent LDL-C reduction and modest HDL-C decrease (Zhang, 2021; Chen et al., 2024; Adam et al., 2020; Wang et al., 2015; Yasuda et al., 2010; Hooper and Burnett, 2013). These mechanistic distinctions provide a biological rationale for target-specific therapy selection.

### 4.3 Comparison with prior evidence

Previous meta-analyses confirmed robust triglyceride and remnant lipoprotein reductions across RNAi therapies but often pooled interventions as a class without target-specific evaluation (Kamrul-Hasan et al., 2024). ASO studies targeting APOC3 showed similar TG-lowering effects with dose-dependent safety considerations (Mahmoud et al., 2025). A broader systematic review of siRNA therapies in dyslipidemia reported heterogeneous lipid effects across targets, but did not provide quantitative comparisons (Alla et al., 2025). Finally, PCSK9-directed siRNA therapy (inclisiran), which predominantly lowers LDL-C with modest triglyceride effects, further illustrates how distinct RNAi targets yield divergent lipid profiles (Khalil et al., 2025). Our analysis incorporates recent ANGPTL3 trials and provides quantitative, target-specific comparisons, highlighting distinct lipid profiles and supporting phenotype-guided therapy.

### 4.4 Clinical implications

The present findings support a target- and phenotype-guided use of siRNA therapies in patients with hypertriglyceridemia and mixed dyslipidemia. In individuals with isolated hypertriglyceridemia, low HDL-C, and LDL-C already at target, APOC3-directed agents (olezarsen and plozasiran) may be preferentially considered, as they provide marked triglyceride lowering while preserving LDL-C control. In contrast, for patients with mixed dyslipidemia requiring simultaneous reduction of triglycerides and apoB-containing lipoproteins, ANGPTL3-targeting therapy (zodasiran) may offer a more comprehensive lipid-lowering strategy. These data highlight the potential role of siRNA therapies as precision adjuncts to standard lipid-lowering treatment, enabling individualized management based on dyslipidemic phenotype.

### 4.5 Limitation

This meta-analysis has several limitations. First, conclusions rely on surrogate endpoints such as lipid parameters and apolipoproteins, and their impact on hard outcomes like myocardial infarction or stroke requires confirmation in longer-term, large-scale trials. Second, included participants were mainly from selective clinical trial populations, limiting representation of key subgroups (e.g., non-Caucasian individuals, patients with severe liver or kidney dysfunction, or rare genetic conditions). Third, variations in baseline characteristics, background therapies, and outcome measurements contributed to moderate-to-high heterogeneity. Fourth, our analysis included only studies published in English, which may introduce language bias and overlook relevant evidence reported in other languages. Finally, data on the use of siRNA therapies in routine clinical practice are limited, and trial results may not fully reflect real-world patient populations, comorbidities, or adherence, which constrains the generalizability of our findings.

### 4.6 Prospect of further studies

We recommend that future research should focus on exploring the following areas: (1) Confirming hard clinical outcomes: Conduct long-term, large-scale trials to establish whether these therapies reduce major adverse cardiovascular events and the incidence of acute pancreatitis. (2) Evaluating use in special populations: Assess efficacy and safety in key groups such as individuals with diabetes and those with advanced renal impairment to inform precision use. (3) Advancing personalized therapy: Leverage target-specific differences in lipid effects to enable “target–phenotype matching” based on each patient’s lipid profile.

## 5 Conclusion

siRNA therapies targeting APOC3 and ANGPTL3 effectively reduce triglycerides and atherogenic lipoproteins, with distinct lipid modulation profiles: APOC3-directed agents favor TG reduction and HDL-C increase, while ANGPTL3-directed agents provide broader lipid-lowering, including LDL-C reduction. These therapies are generally well tolerated and represent promising, target-specific options for precision management of hypertriglyceridemia and mixed dyslipidemia.

## Data Availability

The original contributions presented in the study are included in the article/Supplementary Material, further inquiries can be directed to the corresponding author.
