# Correction: Efficacy and safety of small interfering RNA (siRNA) therapies for hypertriglyceridemia and mixed dyslipidemia: an updated systematic review and meta-analysis

**DOI:** 10.3389/fphar.2026.1904536

**Published:** 2026-06-24

**Authors:** Yifan Gao, Yanmin Bai, Xu Mu, Xingxue Pang

**Affiliations:** 1 Dongzhimen Hospital, Beijing University of Chinese Medicine, Beijing, China; 2 Third Department of Cardiology, Dongzhimen Hospital, Beijing University of Chinese Medicine, Beijing, China

**Keywords:** hypertriglyceridemia, meta-analysis, mixed dislipidemia, small interfering RNA, solbinsiran

In the published article, an incorrect Funding statement was provided. The correct Funding statement appears below.

The author(s) declared that financial support was received for this work and/or publication. This work was supported by the China Academy of Chinese Medical Sciences Innovation Fund (No. CI2021A00905) and Beijing Tongzhou District Science and Technology Program Project (Grant No. WS2025050).

The original article has been updated.

