# Supplementary material for: Efficacy and safety of small interfering RNA (siRNA) therapies for hypertriglyceridemia and mixed dyslipidemia: an updated systematic review and meta-analysis
Source: Front Pharmacol. 2026 Jan 26;17:1736821. doi: 10.3389/fphar.2026.1736821 (PMC12883765; doi:10.3389/fphar.2026.1736821)

frontiers

Supplementary Material

1 Supplementary Tables

1.1 Supplementary Table S1: Search Strategies

Database	Search Strategies
**Pubmed**<br><br>**N = 87**	("small interfering RNA"[Title/Abstract] OR siRNA[Title/Abstract] OR "RNA interference"[Title/Abstract] OR "siRNA therapy"[Title/Abstract] OR "Solbinsiran"[Title/Abstract] OR "Olezarsen"[Title/Abstract] OR "Plozasiran"[Title/Abstract] OR "Zodasiran"[Title/Abstract])<br>AND<br>("hypertriglyceridemia"[Title/Abstract] OR "mixed dyslipidemia"[Title/Abstract] OR "dyslipidemia"[Title/Abstract] OR "hyperlipidemia"[Title/Abstract] OR "triglycerides"[Title/Abstract] OR "lipid metabolism"[Title/Abstract])<br>AND<br>("efficacy"[Title/Abstract] OR "effectiveness"[Title/Abstract] OR "clinical trial"[Title/Abstract] OR "treatment outcome"[Title/Abstract] OR "lipid-lowering"[Title/Abstract])<br>AND<br>("safety"[Title/Abstract] OR "adverse events"[Title/Abstract] OR "side effects"[Title/Abstract] OR "safety profile"[Title/Abstract] OR "tolerability"[Title/Abstract])
**Web of Science**<br><br>**N = 106**	TS=("small interfering RNA" OR siRNA OR "RNA interference" OR "siRNA therapy" OR Solbinsiran OR Olezarsen OR Plozasiran OR Zodasiran)<br>TS=("hypertriglyceridemia" OR "mixed dyslipidemia" OR dyslipidemia OR hyperlipidemia OR triglycerides OR "lipid metabolism")<br>AND<br>TS=("efficacy" OR effectiveness OR "clinical trial" OR "treatment outcome" OR "lipid-lowering")<br>AND<br>TS=("safety" OR "adverse events" OR "side effects" OR "safety profile" OR "tolerability")<br>106
**Embase**<br><br>**N = 501**	('small interfering RNA'/exp OR 'siRNA' OR 'RNA interference' OR 'siRNA therapy' OR 'Solbinsiran' OR 'Olezarsen' OR 'Plozasiran' OR 'Zodasiran')<br>AND<br>('hypertriglyceridemia'/exp OR 'hypertriglyceridemia' OR 'mixed

	dyslipidemia' OR 'dyslipidemia' OR 'hyperlipidemia' OR 'triglycerides' OR 'lipid metabolism')<br>AND<br>('efficacy'/exp OR 'efficacy' OR 'effectiveness' OR 'clinical trial' OR 'treatment outcome' OR 'lipid-lowering')<br>AND<br>('safety'/exp OR 'safety' OR 'adverse events' OR 'side effects' OR 'safety profile' OR 'tolerability')
**Cochrane Library**<br><br>**N = 63**	("small interfering RNA" OR siRNA OR "RNA interference" OR "siRNA therapy" OR Solbinsiran OR Olezarsen OR Plozasiran OR Zodasiran)<br>AND<br>("hypertriglyceridemia" OR "mixed dyslipidemia" OR dyslipidemia OR hyperlipidemia OR triglycerides OR "lipid metabolism")<br>AND<br>(efficacy OR effectiveness OR "clinical trial" OR "treatment outcome" OR "lipid-lowering")<br>AND<br>(safety OR "adverse events" OR "side effects" OR "safety profile" OR "tolerability")

1.2 Supplementary Table S2: Additional study characteristics

Trial ID	No of centers	siRNA treatment	Statin use,n(%)	Ezetimibe, n (%)	Fibrate, n (%)	PCSK9 inhibitor, n (%)	Omega-3 fatty acid, n (%)	Diabetes, n(%)	Chronic kidney disease, n(%)
**ARCHES-2 2024**	25	Subcutaneous injections of zodasiran (50, 100, or 200 mg) on day 1 and week 12	196(96.1)	NA	43(21)	2(0.98)	12(5.9)	86(42.2)	17(8.3)
**MUIR 2024**	36	First three cohorts: Subcutaneous injection of plozasiran (10 mg, 25 mg, or 50 mg) on day 1 and at week 12 (quarterly doses). The fourth cohort: Subcutaneous injection of 50 mg of plozasiran on day 1 and at week 24 (half-yearly dose).	323(91.5)	NA	46(13)	8(2.3)	12(3.4)	215(60.9)	41(11.6)
**PROLONG-ANG3 2025**	41	Subcutaneous injection of solbinsiran (100, 400 mg, or 800 mg) on days 0 and 90	196(95.6)	9(4.4)	NA	NA	NA	92(44.9)	NA
**Balance 2024**	29	Olezarsen (50,80mg)or a matching volume of placebo was administered	16(24.2)	NA	30(45.4)	NA	25(37.9)	16(24.2)	NA

		subcutaneously once every 4 weeks for 49 weeks							
**Bridge–TIMI 73a 2024**	24	50 mg olezarsen , 80 mg olezarsen, or placebo once a month.The treatment period was 12 months.	127(82.5)	10(6.5)	25(16.2)	5(3.2)	34(22.1)	105(68.2)	22(14.3)
**ESSENCE–TIMI 73b 2025**	168	50 mg olezarsen , 80 mg olezarsen, or placebo once a month.The treatment period was 12 months.	1087 (80.6)	229(17)	309(23)	62(4.6)	301(22.3)	809(60)	211 (15.6)
**NCT03385239 2022**	41	Olezarsen (10 or 50 mg every 4 weeks, 15 mg every 2 weeks, or 10 mg every week) or saline placebo subcutaneously for 6–12 months	96(84.2)	14(12.3)	NA	9(7.9)	NA	77(67.5)	NA
**SHASTA-2 2024**	74	Received 2 subcutaneous doses of plozasiran (10, 25, or 50 mg) or matched placebo on day 1 and at week 12	154(68)	NA	109(48)	7(3)	48(21.2)	144(64)	NA

1.3 Supplementary Table S3: Meta-Regression Analyses

Variables	Outcomes	*k*	Coefficient	SE	t	*p*-value	95% CI
Target	TG	8	-3.82	7.35	-0.52	0.621	-21.8 to 14.15
	LDL-C	6	13.85	5.76	2.41	0.074	-2.13 to 29.83
	HDL-C	6	61.11	6.96	8.78	0.001	41.77 to 80.44
	Non-HDL-C	7	6.69	5.35	1.25	0.266	-7.06 to 20.45
	apo B	6	3.54	4.91	0.72	0.511	-10.1 to 17.18
Specific siRNA drug	TG	8	-0.05	3.27	-0.02	0.987	-8.07 to 7.96
	LDL-C	6	0.32	5.03	0.06	0.952	-13.63 to 14.28
	HDL-C	6	-23.54	7.37	-3.2	0.033	-44 to -3.09
	Non-HDL-C	7	-3.59	2.21	-1.62	0.165	-9.27 to 2.09
	apo B	6	-1.98	2.05	-0.97	0.387	-7.66 to 3.69

k = Number of trials, SE = Standard error, CI = Confidence interval.

2 Supplementary Figures

2.1 Supplementary Figure S1: Leave-one-out sensitivity analysis of the efficacy and safety outcomes.

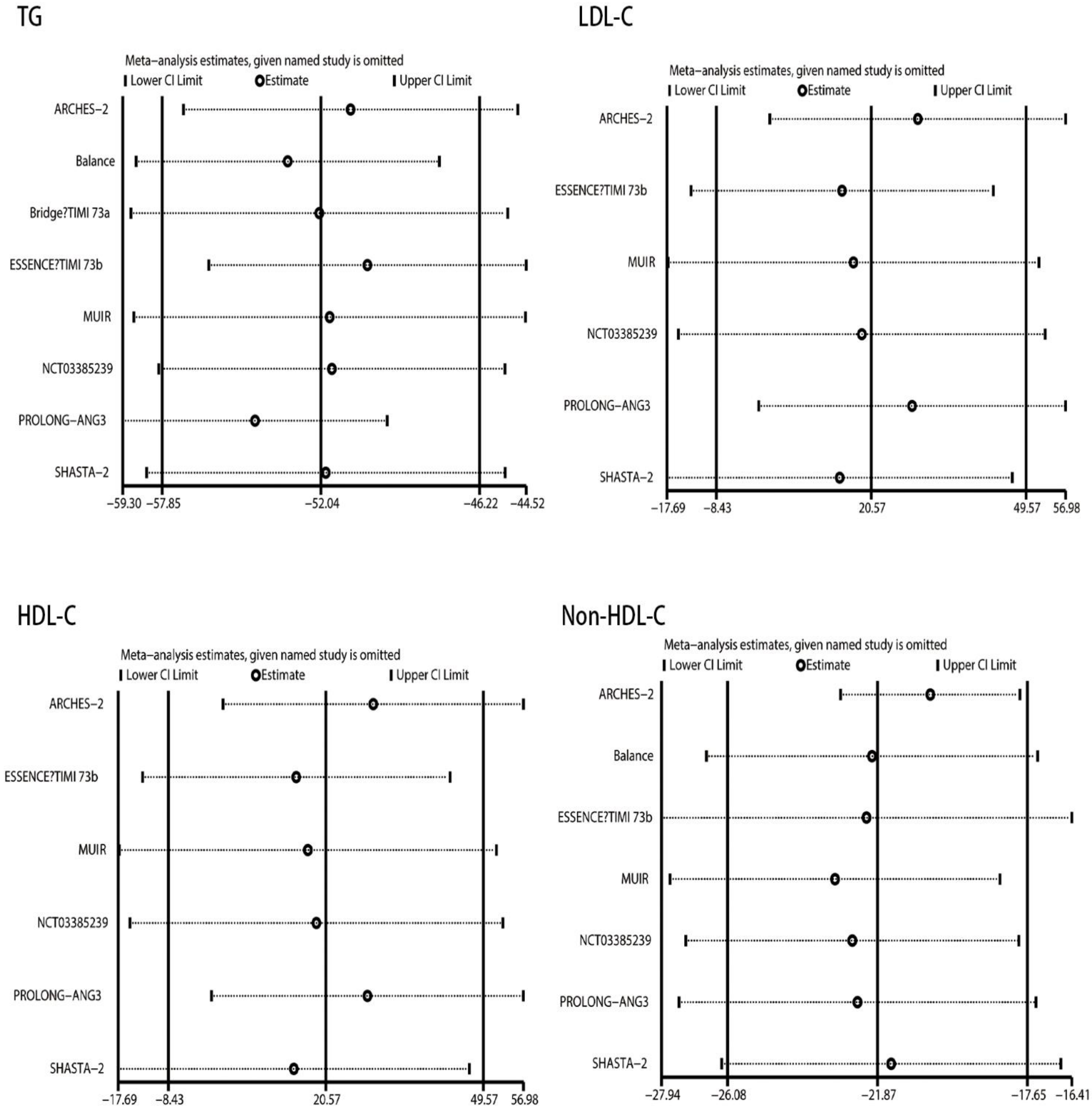

VLDL-C

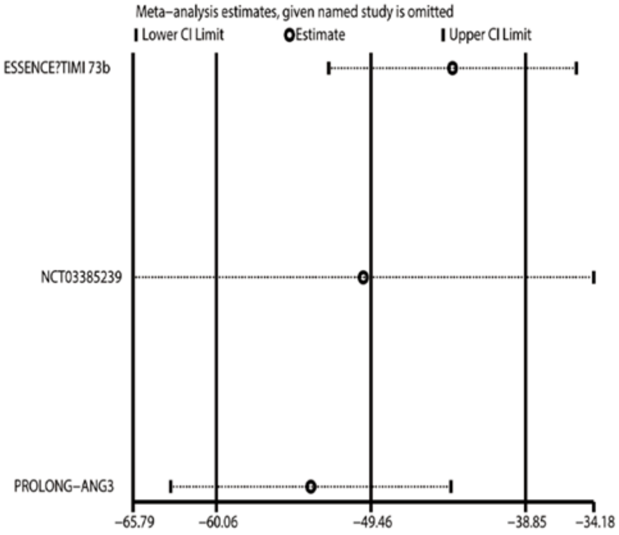

apoB

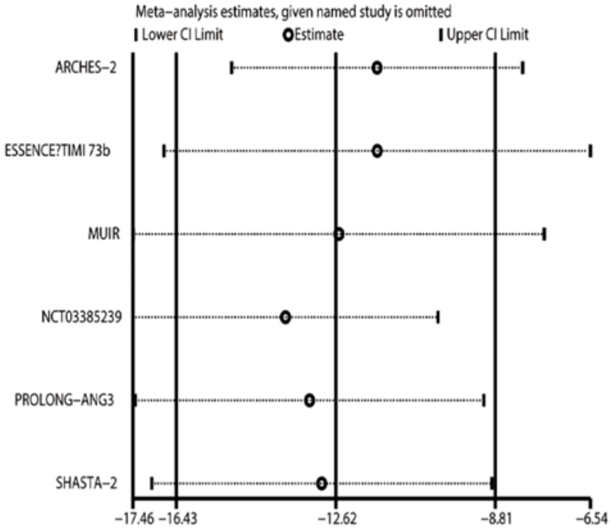

Remnant Cholesterol

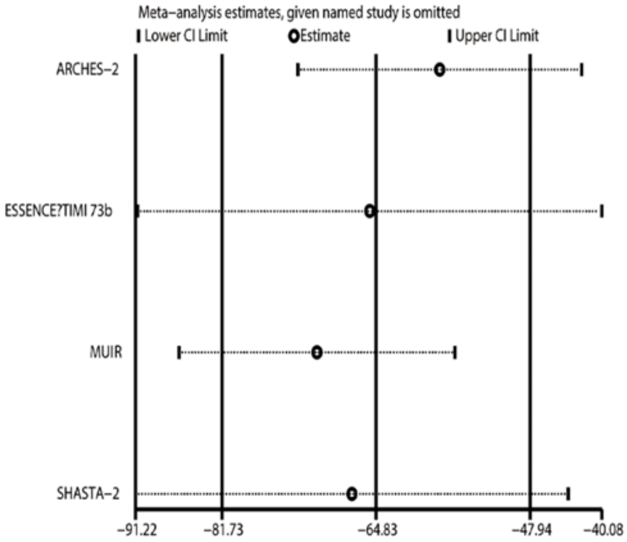

Adverse events

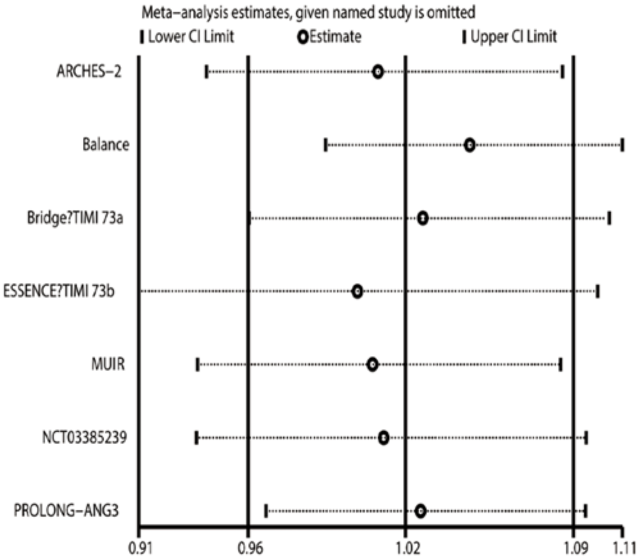

2.2 Supplementary Figure S2: Risk of Bias Assessment for Included Randomized Controlled Trials

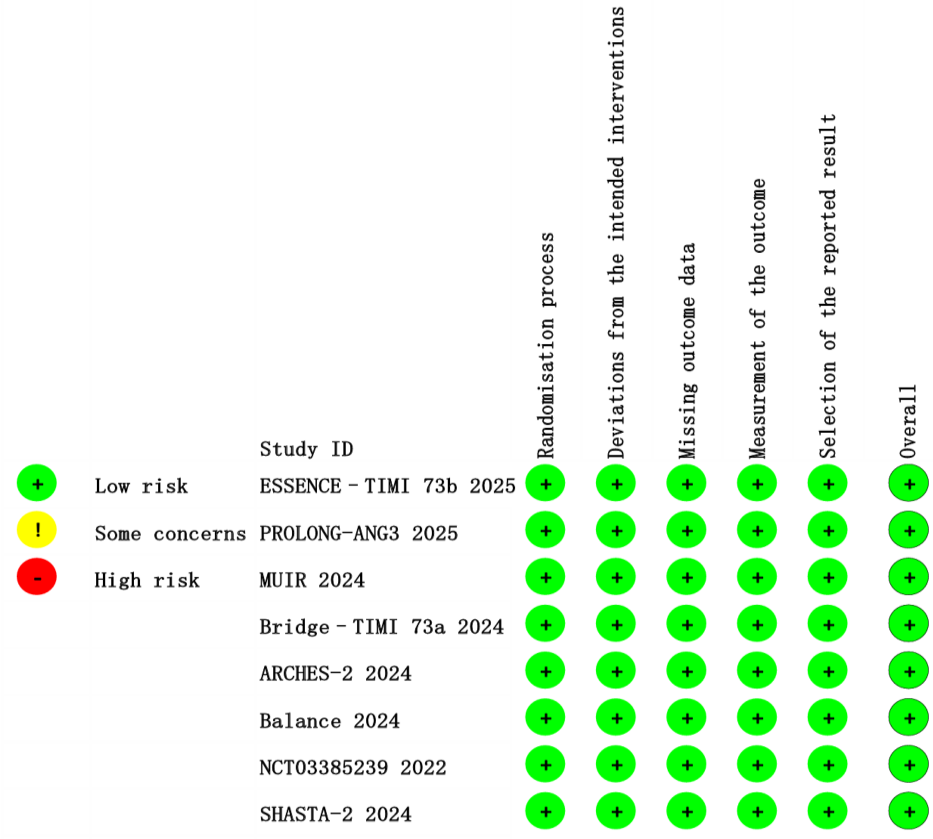

Supplement: Supplementary file 2 [file DataSheet1.pdf]
